# Supplementary material for: Density Distribution Maps: A Novel Tool for Subcellular Distribution Analysis and Quantitative Biomedical Imaging
Source: Sensors (Basel). 2021 Feb 2;21(3):1009. doi: 10.3390/s21031009 (PMC7867329; doi:10.3390/s21031009)
Supplement: Supplementary file 1 [file sensors-21-01009-s001.pdf]

sensors

MDPI

Supplementary Materials

Density distribution maps: a novel tool for subcellular distribution analysis and quantitative biomedical imaging

Ilaria De Santis [1,2], Michele Zanoni [3], Chiara Arienti [3], Alessandro Bevilacqua [4,5,*], Anna Tesei [3]

[1] Department of Medical and Surgical Sciences (DIMEC), Alma Mater Studiorum, University of Bologna, I-40138 Bologna, Italy; i.desantis@unibo.it
[2] Interdepartmental Centre Alma Mater Research Institute on Global Challenges and Climate Change (Alma Climate), University of Bologna, I-40126 Bologna, Italy
[3] Biosciences Laboratory, IRCCS Istituto Scientifico Romagnolo per lo Studio e la Cura dei Tumori (IRST) "Dino Amadori", I-47014 Meldola, Italy; michele.zanoni@irst.emr.it (M.Z.); chiara.arienti@irst.emr.it (C.A.); anna.tesei@irst.emr.it (A.T.)
[4] Advanced Research Center on Electronic Systems (ARCES) for Information and Communication Technologies "E. De Castro", University of Bologna, I-40125 Bologna, Italy
[5] Department of Computer Science and Engineering (DISI), University of Bologna, I-40136 Bologna, Italy
* Correspondence: alessandro.bevilacqua@unibo.it; Tel.: +39-051-20-9-5409

Table 1. Median MTs intensity in MG-63 cells.

	Median intensity ± MAD	
**Optical section**	**CTR (n=17)**	**PTX-Ce6@-ker$_{ag}$ (n=14)**
1	24.0±5.00	782±197
2	36.0±8.00	918±233
3	53.0±11.0	991±250
4	76.0±17.0	920±236
5	123±27.0	781±193
6	210±46.0	683±163
7	347±76.0	752±195
8	460±105	717±180
9	525±128	828±216
10	563±143	857±236
11	555±148	862±237
12	553±147	789±214
13	524±141	726±198
14	460±124	663±181
15	377±103	-
16	277±72.0	-
17	199±50.0	-

Table 2. Statistical analysis of MTs LDI percentage in MG-63 cells.

	Median percentage (%) ± MAD		
**LDI**	**CTR (n=17)**	**PTX-Ce6@-ker$_{ag}$ (n=14)**	**p-value (<0.05)**
0	1.40±0.96	0.05±0.01	$<10^{-5}$
1	2.15±1.26	0.10±0.02	$<10^{-5}$
2	3.01±1.53	0.31±0.03	$<10^{-5}$
3	4.54±1.87	0.96±0.06	$<10^{-5}$
4	5.94±1.05	3.13±0.16	$<10^{-5}$
5	8.15±1.08	5.88±0.38	$<10^{-3}$
6	9.27±0.79	4.07±0.16	$<10^{-5}$
7	12.4±0.79	5.84±0.33	$<10^{-3}$
8	47.1±14.2	79.5±0.84	$<10^{-5}$

Table 3. Statistical analysis of hybrids LDI percentage in A549 sh/p53 cells.

LDI	Nucleus<br>Median percentage (%) ± MAD<br>CTR (n=14)	2 Gy (n=14)	p-value (<0.05)	Cytoplasm<br>Median percentage (%) ± MAD<br>CTR (n=14)	2 Gy (n=14)	p-value (<0.05)
0	0.32±0.13	1.63±0.97	$<10^{-4}$	0.44±0.12	1.86±0.60	$<10^{-5}$
1	0.88±0.26	2.39±1.65	$<10^{-3}$	0.95±0.28	3.02±0.87	$<10^{-5}$
2	1.81±0.42	3.84±1.74	$<10^{-2}$	2.89±0.40	4.79±0.93	$<10^{-5}$
3	4.24±0.73	6.07±2.45	0.02	7.85±0.98	9.06±1.29	0.05
4	7.50±1.11	10.0±3.01	0.08	13.4±1.17	12.1±1.42	0.07
5	9.91±1.05	11.5±1.83	0.05	14.9±0.89	13.4±0.95	$<10^{-2}$
6	10.6±1.03	11.0±1.37	0.30	12.2±0.38	12.6±1.03	0.73
7	13.6±1.02	13.1±1.71	0.80	15.0±0.38	14.2±0.99	$<10^{-2}$
8	51.1±5.05	41.3±12.7	0.01	32.0±3.14	28.0±6.29	0.07

Table 4. Median hybrids LDI percentage and blobs number in HeLa cells.

LDI	Median percentage (%) ± MAD<br>CTR (n=5)	Cortical (n=4)	Intermediate (n=3)	Scattered (n=3)	(Median blob number (pixel^{-1}) ± MAD) *10^{-3}<br>CTR (n=5)	Cortical (n=4)	Intermediate (n=3)	Scattered (n=3)
0	0.06±0.03	0.20±0.03	0.15±0.01	0.16±0.04	0.11±0.05	0.44±0.07	0.32±0.01	0.36±0.08
1	0.30±0.04	0.51±0.03	0.49±0.02	0.48±0.01	0.38±0.01	0.76±0.04	0.67±0.05	0.67±0.14
2	0.58±0.10	1.27±0.11	1.18±0.05	1.25±0.05	0.85±0.20	2.43±0.24	1.99±0.13	2.15±0.05
3	2.62±0.14	4.21±0.17	4.50±0.56	5.01±0.29	4.57±0.55	8.12±0.07	8.75±0.20	10.10±0.12
4	10.30±0.78	10.30±0.75	11.70±0.78	12.40±0.57	17.6±1.54	19.60±1.34	21.00±0.88	23.80±0.38
5	12.50±1.02	14.10±0.32	15.30±1.12	15.64±0.44	18.00±1.46	20.90±0.51	22.50±0.52	25.00±0.08
6	7.26±0.16	9.12±0.25	9.24±0.07	9.51±0.26	10.70±0.77	15.80±0.09	16.20±0.76	16.90±0.13
7	13.20±0.58	12.60±0.32	13.50±0.56	14.28±0.14	19.50±1.99	21.40±0.49	22.30±0.96	24.50±0.41
8	53.00±2.99	48.3±1.05	40.00±4.11	41.68±1.70	2.63±0.10	2.54±0.08	3.79±0.36	3.71±0.44

Table 5. Statistical analysis of hybrids LDI percentage and blob number in HeLa cells.

LDI	p-value (<0.05)<br>LDI percentage (%)<br>CTR-All	CTR-Cortical	CTR-Intermediate	CTR-Scattered	Blob number (pixel^{-1})<br>CTR-All	CTR-Cortical	CTR-Intermediate	CTR-Scattered
0	0.01	0.02	0.14	0.14	0.26	0.19	0.99	0.32
1	$<10^{-2}$	0.02	0.14	0.07	0.24	0.22	0.50	0.68
2	$<10^{-2}$	0.02	0.04	0.04	0.38	0.76	0.79	0.25
3	$<10^{-2}$	0.02	0.04	0.03	0.37	0.03	0.57	0.39
4	0.31	0.73	0.25	0.07	0.01	0.02	0.07	0.25
5	0.03	0.41	0.07	0.04	0.01	0.02	0.07	0.25
6	$<10^{-2}$	0.02	0.04	0.04	0.03	0.02	0.07	0.79
7	0.31	0.90	0.57	0.04	$<10^{-2}$	0.02	0.04	0.07
8	$<10^{-2}$	0.06	0.04	0.04	0.01	0.02	0.07	0.29

Table 6. Variance of hybrids LDI percentage and blob number in HeLa cells.

	Variance							
	**LDI percentage (%)**				**Blob number (pixel^{-1}) *10^{-7}**			
**LDI**	**All (n=10)**	**Cortical (n=4)**	**Intermediate (n=3)**	**Scattered (n=3)**	**All (n=10)**	**Cortical (n=4)**	**Intermediate (n=3)**	**Scattered (n=3)**
0	0.003	0.002	0.002	0.004	0.15	0.09	0.10	0.17
1	0.01	0.002	0.01	0.01	0.09	0.05	0.03	0.22
2	0.03	0.05	0.04	0.03	1.23	1.94	0.24	0.66
3	0.32	0.14	0.67	0.10	11.2	3.16	14.6	3.02
4	1.44	0.89	1.02	0.40	41.1	29.7	9.36	16.4
5	1.48	0.35	2.61	0.50	35.4	30.9	21.3	11.5
6	0.12	0.21	0.05	0.08	7.38	6.58	10.5	1.88
7	0.76	0.75	0.67	0.04	21.0	16.2	10.4	9.06
8	15.8	9.85	21.4	4.94	4.42	2.17	3.19	2.08

Table 7. Abbreviations.

**Ab**	**Antibody**
**CT**	Computed Tomography
**CTR**	Control
**CV**	Coefficient of Variation
**DMM**	Density Distribution Map
**FG**	Foreground
**FN**	False Negative
**FNR**	False Negative Rate
**FP**	False Positive
**GUI**	Graphical User Interface
**LDI**	Local Density Index
**MAD**	Median Absolute Deviation
**MIP**	Maximum Intensity Projection
**MT**	Microtubule
**NCC**	Normalized Cross Correlation
**PTX**	Paclitaxel
**SDR**	Stoichiometric Detection Rate
**SE**	Structuring Element
**SMLM**	Single-Molecule Localization Microscopy
**TN**	True Negative
**TP**	True Positive
**TPR**	True Positive Rate
**V2R**	Vasopressin Receptor 2
